# Cardiac parameter estimation from electrocardiograms based on inversion of physical simulator behavior

**DOI:** 10.1371/journal.pdig.0001624

**Published:** 2026-08-07

**Authors:** Ryo Nishikimi, Yoshifumi Shiraki, Shingo Tsukada, Kunio Kashino

**Affiliations:** 1 Communication Science Laboratories, NTT, Inc., Atsugi-shi, Kanagawa, Japan; 2 Biomedical Informatics Research Center, NTT, Inc., Atsugi-shi, Kanagawa, Japan; National University of Singapore, SINGAPORE

## Abstract

Electrocardiograms (ECGs) are used in clinical medicine because their waveforms provide rich information reflecting heart states. It is believed that a specialist with extensive training can identify representative abnormalities and their predictive signs through template matching with typical cases. Furthermore, it is increasingly required to identify the cause of the abnormality, which is difficult even for specialists. To provide clues to internal heart states, we propose a deep neural network-based model composed of one encoder network and multiple decoder networks for converting a 12-lead ECG into multiple microscopic physical and chemical cardiac parameters that represent internal heart states and are potentially essential factors of ECG signals, such as multiple ionic parameters related to cardiac electrophysiology. Data pairs consisting of cardiac parameters and ECGs are needed for training the model, but these are unavailable in principle. Therefore, we propose to generate such data by using a white-box model that can simulate the physical behavior of the heart. The proposed method was experimentally evaluated in terms of the mean absolute error of cardiac parameters taking continuous values and in terms of the accuracy of those taking discrete values. The results showed that the method could accurately estimate these parameters. Moreover, we investigated the estimation errors in detail by visualizing the distributions of the estimated cardiac parameters around the ground-truth values. Our method can help doctors monitor heart conditions by automatically estimating cardiac parameters from ECGs. In addition, it can be used to discover relationships among cardiac parameters, ECG waveforms, and heart diseases. However, before our method can be used in actual clinical practice, it remains to be verified that it works correctly with real ECGs.

## Author summary

This study is a novel attempt to develop a deep neural network method that, in combination with a sophisticated heart simulator, can simultaneously estimate multiple microscopic cardiac factors of ECGs. The proposed model is trained using data synthesized by a heart simulator. We experimentally confirmed that our method can accurately estimate multiple factors based on evaluation metrics (mean absolute error and accuracy). This research opens the door to the possibility of a practical and comprehensive analysis of microscopic cardiac factors of ECG signals. In particular, the factors estimated from ECGs enable us to understand the internal states of the heart that would otherwise be difficult to ascertain only from the appearance of the ECG. They are also useful for discovering unknown relationships between cardiac parameters and ECGs. Thus, our method will contribute to early diagnosis and medical progress. In addition, since our method is based on a general framework of deep neural networks, our approach can be used on other organs and factors. As part of our future work, we will verify whether our method works correctly with real ECGs, and we plan to consult with medical experts to ensure that it can be used in actual clinical practice.

## Introduction

An electrocardiogram (ECG) is a graphical representation of the electric potential changes produced by the excitation of myocardial cells, and they are detected using electrodes placed at specific locations on the body [1–3]. ECGs are especially useful for monitoring cardiac health and detecting heart disease in clinical settings [4–6]. Doctors generally diagnose heart conditions by categorizing the morphological patterns of the patient’s ECG waveform in accordance with their own experience and past cases. However, visual diagnosis may overlook trivial signs of cardiac abnormalities in the ECG waveform. Therefore, a fast and accurate method that can automatically analyze and diagnose the internal state of the heart would be helpful in clinical practice.

Many studies have attempted to analyze ECGs by using data-analysis and machine-learning techniques. Classification problems that involve predicting the categories of heart states such as arrhythmias, myocardial infarctions, risk of mortality, atrial fibrillation, and ventricular dysfunction have been actively studied [7–16]. The methods that have been developed in these studies are helpful for identifying heart states, but they can estimate only predefined categories of cardiac abnormalities. If new cardiac abnormality categories become known as medical science advances, these methods would have to be updated or their models retrained to accommodate them. In particular, such updating or retraining requires new labeled datasets corresponding to the new categories, which are time-consuming to create.

To address this situation, we believe that it is necessary to estimate the microscopic factors themselves at the cellular level that are potentially essential factors of ECG signals in the human body, instead of the artificially predefined categories. If it becomes possible to estimate the microscopic generative factors of ECGs, they can be useful clues for indirectly estimating cardiac abnormality categories. For example, Type 1 long QT syndrome (LQT1), characterized by reduced I_Ks_, can trigger fatal arrhythmias during high-intensity exercise like swimming or marathon running. In contrast, Type 2 long QT syndrome (LQT2), characterized by reduced I_Kr_, can trigger fatal arrhythmias due to bradycardia during rest. When genetic testing cannot be performed for any reason, or when genetic testing fails to identify an abnormality, distinguishing between I_Ks_ (LQT1) and I_Kr_ (LQT2) via ECG enables lifestyle guidance to avoid triggers of fatal arrhythmias. Several studies have developed methods for solving classification and regression problems related to such generative factors of ECGs, especially electrolytes [17–25]. However, these studies focus on estimating the concentrations of substances such as potassium [17,20,22,24,25], sodium [17], calcium [17,25], and creatinine [17], or on detecting anomalies like hyperkalemia [20,23] and hypokalemia [20] based on classification. As described in a later section, the focus of these studies differs from the generative factors of ECGs that we aim to estimate (Table 1). Moreover, these existing studies can deal with only a limited range of factors. Currently, no dataset comprehensively covers a wide range of generative factors of ECGs and can be used to develop such methods, and manually creating such a dataset is time-consuming. Therefore, as a first step towards alleviating the lack of data covering a wide range of generative factors of ECGs, we decided to use methods that can synthesize ECG signals.

**Table 1:** Cardiac parameters.

Name	Description
I_Kr_	Conductance of a rapid component of delayed rectifier outward K^+^ current. It takes two continuous values: 100% or 70%.
I_Ks_	Conductance of a slow component of delayed rectifier outward K^+^ current. It takes two continuous values: 100% or 60%.
I_to_	Conductance of transient outward K^+^ current. It takes two continuous values: 100% or 60%.
I_K1_	Conductance of inward rectifier K^+^ current. It takes two continuous values: 100% or 70%.
I_Na_, I_NaL_	Conductance of fast and late Na^+^ current. The tuple of currents takes two continuous values: (100%, 100%) or (80%, 200%).
I_CaL_	Conductance of L-type Ca^2 +^ current. It takes two continuous values: 100% or 80%.
NCX	Activity of Na ^+^ -Ca^2 +^ exchanger current. It takes two continuous values: 100% or 200%.
SERCA	Activity of calcium uptake via sarco/endoplasmic reticulum Ca^2 +^ ATPase pump. It takes two continuous values: 100% or 80%.
MK	Fraction of active Ca^2 +^ /calmodulin-dependent protein kinase II (CaMK) binding sites at equilibrium and Ca^2 +^ leakage from the network sarcoplasmic reticulum (NSR). It takes two continuous values: 100% or 150%.
G_fib_, G_Cross_	Conduction velocity in fiber/cross-fiber directions. When LV represents normal or dilated cases, the tuple takes two continuous values: (100%, 100%) or (120%, 80%). When LV represents the hypertrophic case, the tuple takes two continuous values: (100%, 100%) or (130%, 100%).
LV	Sphericity index quantifying the left ventricular morphology. It takes three discrete patterns corresponding to the normal (SI=0.53), delated (SI=0.68), and hypertrophic (SI=0.48) cases, where SI stands for the sphericity index defined by (sphericity index) = (heart long-axis length) / (heart diameter).
EX	Ventricular activation pattern. It takes five discrete patterns: normal, left axis deviation, right axis deviation, left anterior fascicular block, and incomplete right bundle branch block.
CELL	Arrangement pattern of the three ventricular cell models, i.e., endocardial cell, M-cell, and epicardial cell. It takes two discrete patterns.

There are some methods of synthesizing ECGs. The ones based on dynamical models are composed of ordinary differential equations representing the shape of the ECG waveform [26]. Moreover, there are ones based on deep neural networks (DNNs) [27–29], and there is also an ECG simulator called UT-Heart [30,31] that is based on the finite element method (FEM); it can synthesize 12-lead ECG signals from physical and chemical parameters, including electrochemical ones at the cellular level. The input parameters of UT-Heart exactly match the generative factors of ECGs that we aim to estimate. Therefore, in this paper, we will refer to the input parameters of UT-Heart, which consist of 15 types listed in Table 1, as cardiac parameters, and we will use them together with ECGs synthesized by UT-Heart as output and input data for the task at hand.

This study proposes a method for simultaneously estimating multiple cardiac parameters from 12-lead ECG signals. The proposed model is trained using both the inputs and outputs of a physical simulator, specifically the cardiac parameters and the corresponding ECG signals. This task is challenging because it requires the model to learn how to simulate the inverse of the complex ECG generation process. In general, inverse problems are challenging [32] and often cannot be solved analytically. Therefore, even with the physical heart model, the inverse problem of estimating the cardiac parameters is not necessarily easy to solve. To solve this problem, we developed a deep neural network (DNN) consisting of one encoder and multiple decoders. Experimental results demonstrate that the proposed method can accurately estimate the cardiac parameters.

## Materials and methods

### Dataset

We used an ECG dataset created by the multiscale multiphysics heart simulator, UT-Heart [30,31]. All ECGs were sampled at 500 Hz. Since the heart rate was fixed at 60 beats per minute, the duration of one beat was one second. Although every ECG recording was ten seconds long (i.e., ten beats), the waveforms were identical across beats. Therefore, we used only a one-second segment corresponding to one beat for each data. UT-Heart can synthesize an ECG signal from 15 physical and chemical parameters listed in Table 1, including electrochemical parameters at the cellular level. These parameters are employed to determine the full-scale behavior of the heart within the UT-heart simulator [30,31]. Thus, we will refer to them as cardiac parameters and develop a model that predicts all of them from the ECG. Thirteen of the cardiac parameters take continuous values and the other two take discrete ones, so we will refer to them particularly as continuous cardiac parameters and discrete cardiac parameters. Each continuous parameter except for LV is associated with one normal and one abnormal pattern. The dataset contains 30720 variations of pairs of the 12-lead ECG and 15 cardiac parameters, corresponding to the number of combinations of the above cardiac parameters. More detailed information, including the meanings of each cardiac parameter, is described on the website [33]. Additionally, the validation and discussion of the synthetic ECGs can be found in [30,31].

The proposed model outputs the cardiac parameters used as input for the conventional FEM-based method. The parameter names are listed in the left column, and their descriptions are in the right column. EX and CELL are discrete parameters, and the others are continuous parameters. A value of 100% for the continuous parameters means normal.

The ECGs and cardiac parameters in the dataset were transformed so that the proposed model can take them as input and output data. The amplitude of the ECGs was divided by 1024 to make its absolute value between zero and one. Other signal processing techniques, such as noise reduction and filtering, are not applied to the ECG signals prior to model training. The continuous cardiac parameters, except for the LV parameter, were divided by 200 so that their values would fall within the range of zero to one. The LV parameter was not transformed because its value is between zero and one by default. Each discrete cardiac parameter was converted into a one-hot vector. Specifically, a discrete cardiac parameter taking the *j*-th value among *L* values is represented as an *L*-dimensional vector where only the *j*-th element is one and the others are zero. The continuous parameters take only a limited number of distinct values, which makes them appear discrete. However, since they represent continuous quantities defined as percentages rather than discrete categorical quantities, we treat them as continuous variables.

### Model

We developed a deep-learning model for estimating the cardiac parameters from 12-lead ECG signals. The model takes one cycle of a 12-lead ECG signal $𝐗=({𝐱}_{1},...,{𝐱}_{T})\in {\mathbb{R}}^{12\times T}$ as input and outputs a transformed cardiac parameter vector $𝐘=({𝐲}_{1},...,{𝐲}_{15})$, where *T* is the number of samples per ECG cycle, ${𝐱}_{t}=(x_{t,1},...,x_{t,12})\in {\mathbb{R}}^{12}$ is a 12-dimensional vector of ECG values at sample t∈{1,...,T}, and ${𝐲}_{i}\;(i\in \{1,...,15\})$ represents the transformed cardiac parameter. Here, let C={1,...,13} and *D*={14,15} be the index sets indicating the continuous and discrete cardiac parameters, respectively, the transformed continuous cardiac parameter ${𝐲}_{i}\;(i\in C)$ is a scalar value and the transformed discrete cardiac parameter ${𝐲}_{i}\;(i\in D)$ is a vector of size $L_{i}$, i.e., ${𝐲}_{i}=(y_{i,1},...,y_{i,L_{i}})$. To keep the notation consistent, we set $L_{i}=1\;(i\in C)$ for the continuous cardiac parameters. Note that we denote the ground-truth transformed cardiac parameters as ${𝐘}^{*}=({𝐲}_{1}^{*},...,{𝐲}_{15}^{*})$ to distinguish them from the output of the model 𝐘.

**Fig 1:**
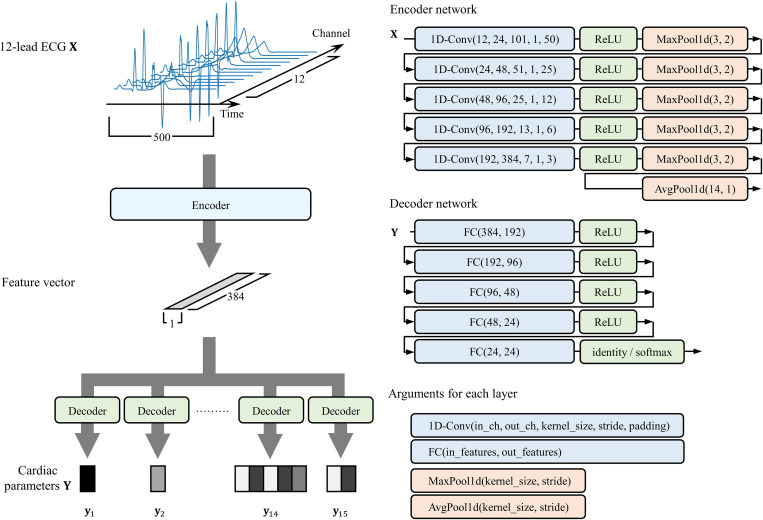
Overview of the network architecture of the proposed model. The proposed model is based on a DNN composed of an encoder network and a decoder network, which are a stack of one-dimensional convolutional layers and fully connected layers. The blue, green, and red boxes in the right figures represent data transformation layers with learnable parameters, activation function layers, and pooling layers, respectively. The transformation and pooling layers take several arguments, which are described in the box at the lower right of the figure. The proposed model is implemented with PyTorch 2.5.1, [34], and default values are used for the arguments not shown in the figure.

We empirically determined the network architecture of the proposed model (Fig 1) based on the results of several preliminary experiments. In these experiments, a baseline model, which is an encoder of convolutional layers followed by a single decoder of fully connected layers, showed inferior parameter-estimation performance (Table 2). Therefore, we decided to use decoders corresponding to individual parameters. The model is based on a DNN composed of an encoder network followed by multiple decoder networks. The encoder network outputs a fixed-size vector representing the feature of the entire 12-channel ECG signal. By having one encoder network for all cardiac parameters, we expect that the output feature vector can explain the interrelationships between the cardiac parameters. While we use only one encoder network, each cardiac parameter has its dedicated decoder network. We expected that each decoder would learn the unique characteristics of each cardiac parameter and thereby improve the accuracy of the cardiac parameter prediction.

**Table 2:** An example of preliminary experiments.

Model	I_Kr_	I_Ks_	I_to_	I_K1_	I_Na_	I_NaL_	I_CaL_	NCX	SERCA	MK	G_Fib_	G_Cross_	LV	EX	CELL
Proposed	0.06	0.14	0.40	0.07	0.17	0.04	0.16	0.17	0.98	2.13	0.20	0.05	0.00	99.87	100.00
Baseline	0.16	14.88	0.37	0.14	0.05	0.31	7.33	0.79	6.94	8.83	0.23	0.12	0.00	99.84	100.00

The encoder network is composed of five one-dimensional convolutional (1D-Conv) layers, which is a popular choice in ECG analysis [10]. The first 1D-Conv layer has 12 channels corresponding to the number of ECG leads. We set the output channel size of each 1D-Conv layer to twice the input channel size. In addition, the stride and padding sizes of each 1D-Conv layer are set so that the length of the input sequence is the same as that of the output sequence. We use a rectifier linear unit (ReLU) as an activation function, and the outputs of the ReLU activation layers are passed to the max-pooling layers. Finally, the output of the last max-pooling layer is averaged along the time axis to obtain a fixed-size feature vector that is passed to the decoder networks.

In the preliminary experiments, we compare two models: the baseline model and the proposed model. The baseline model consists of an encoder composed of convolutional layers, followed by a single decoder composed of fully connected layers. The proposed model uses the same encoder followed by multiple decoders corresponding to individual cardiac parameters. In both models, the decoders share the same architecture, except for the output size of the final layers. The evaluation metrics are the mean absolute error (Eq (7)) for the continuous cardiac parameters and the accuracy (Eq (8)) for the discrete cardiac parameters.

The model has twelve decoder networks corresponding to individual cardiac parameters, and they take the fixed-size feature vector obtained from the encoder and output the transformed cardiac parameters. Each decoder network is composed of five fully connected (FC) layers, and the output size of each FC layer, except the last one, is set to half the input size. The output size of the last FC layer of each decoder network is determined by the size of the transformed cardiac parameter corresponding to the decoder network. Specifically, the output size of the decoder networks is $L_{i}\;(i\in C\cup D)$. ReLU is used as an activation function of the FC layers except for the last FC layer of each decoder network. The last activation function of the decoder networks corresponding to the continuous cardiac parameters is an identity function, while that of the decoder networks corresponding to the discrete cardiac parameters is a softmax function, defined as


$$\begin{array}{c} {𝐲}_{i}=Softmax({𝐳}_{i})={\left(\frac{\exp z_{i,j}}{\sum_{k=1}^{L_{i}}\exp z_{i,k}}\right)}_{j=1,...,L_{i}}(i\in D), \end{array}$$ (1)

where ${𝐳}_{i}=(z_{i,1},...,z_{i,L_{i}})$ is the output of the last FC layer of the decoder network corresponding to the discrete cardiac parameter indexed by *i*. The hyperparameter settings of the layers in the proposed model are described in detail in Fig 1.

### Training

We trained the proposed model with the data of the 12-lead ECGs 𝐗 and their corresponding cardiac parameters ${𝐘}^{*}$. Specifically, we used the stochastic gradient descent method to gradually update the parameters of the 1D-Conv and FC layers by minimizing the error between the transformed cardiac parameters ${𝐘}^{*}$ in the ground-truth dataset and those 𝐘 estimated by the proposed method from the 12-lead ECG 𝐗. To quantify the error, we used a loss function expressed as a sum of 15 terms as follows:


$$\begin{array}{c} ℒ\left(𝐘,{𝐘}^{*}\right)=\frac{1}{M}\sum_{m=1}^{M}\left(\sum_{i=1}^{13}{ℒ}_{C}\left(y_{m,i},y_{m,i}^{*}\right)+\sum_{i=14}^{15}{ℒ}_{D}\left({𝐲}_{m,i},{𝐲}_{m,i}^{*}\right)\right) \end{array}$$ (2)

where *M* is the number of the data in a mini-batch, ${𝐘}_{m}^{*}=(y_{m,1}^{*},...,y_{m,13}^{*},{𝐲}_{m,14}^{*},{𝐲}_{m,15}^{*})$ is the *m*-th set of ground-truth transformed cardiac parameters in the mini-batch, ${𝐘}_{m}=(y_{m,1},...,y_{m,13},{𝐲}_{m,14},{𝐲}_{m,15})$ is the set of transformed cardiac parameters estimated by the proposed model from the *m*-th 12-lead ECG ${𝐗}_{m}$ in the mini-batch, ${ℒ}_{C}$ is the loss function that calculates the errors of the continuous cardiac parameters, and ${ℒ}_{D}$ is for the discrete cardiac parameters. In particular, for the continuous cardiac parameters, we chose L1 loss to ensure consistency with the absolute error metric used to evaluate their estimation results. The definition of the L1 loss function is given as


$$\begin{array}{c} {ℒ}_{C}\left(y_{m,i},y_{m,i}^{*}\right)=\left|y_{m,i}-y_{m,i}^{*}\right|\left(i\in C\right) \end{array}$$ (3)

For the discrete cardiac parameters, we used the cross-entropy function, which is one of the most popular loss functions for classification tasks:


$$\begin{array}{c} {ℒ}_{D}\left({𝐲}_{m,i},{𝐲}_{m,i}^{*}\right)=-\sum_{j=1}^{L_{i}}y_{m,i,j}^{*}\log y_{m,i,j}\left(i\in D\right) \end{array}$$ (4)

### Inference

We can obtain original cardiac parameters $\Phi =(\phi_{i}{)}_{i=1}^{15}$ from the output of the proposed model 𝐘 by performing the inverse operation of the cardiac parameter transformation described in the dataset subsection. Here, each original continuous cardiac parameter $\phi_{i}\;(i\in C)$, except the LV parameter, is obtained by multiplying $y_{i}$ by 200


$$\begin{array}{c} \phi_{i}=200y_{i}. \end{array}$$ (5)

Since the LV parameter is not transformed before it is used to train the model, we can use the output of the proposed model directly as a predictor of the LV parameter. Each original discrete cardiac parameter $\phi_{i}\;(i\in D)$ is obtained by taking the index *j* that gives the maximum value of $\left(y_{i,1},...,y_{i,L_{i}}\right)$:


$$\begin{array}{c} \phi_{i}=\underset{j\in \{1,...,L_{i}\}}{argmin}\;y_{i,j} \end{array}$$ (6)

### Experimental conditions

**Fig 2:**
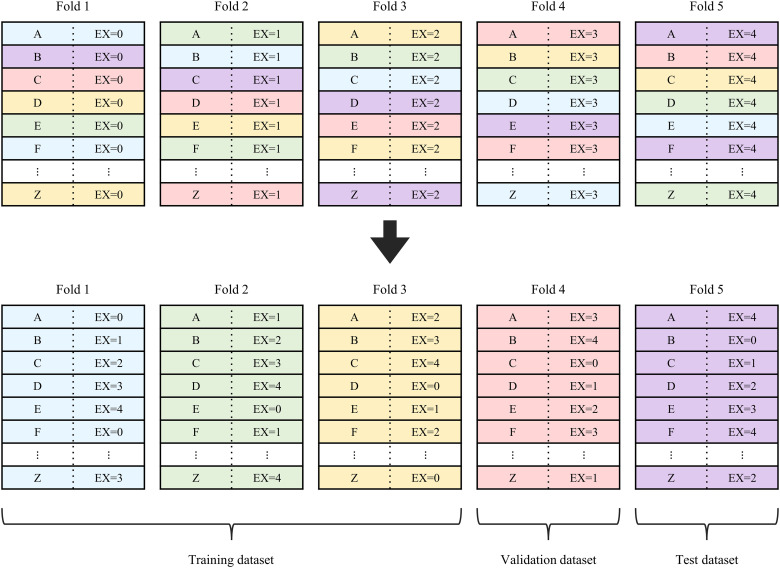
Dataset creation procedure. The training, validation, and test datasets used for the proposed method are created by splitting and shuffling the original dataset. The upper tables represent the original data folds, and the lower tables represent the data folds obtained through a cyclic data shuffle indicated by the five colors.

We split the 30720 data synthesized by UT-Heart into a training dataset (18432 data), a validation dataset (6144 data), and a test dataset (6144 data) at a ratio of 60:20:20. To keep the number of data samples balanced across cardiac parameter values for each of the training, validation, and test sets, as shown in Fig 2, each dataset was created by cyclically shuffling the original dataset to contain multiple EX parameter values. Initially, the original dataset was divided into five folds based on the values of the EX parameter. In other words, each fold initially contained only one EX parameter value. Each fold included all combinations of cardiac parameters except for the EX parameter, and Fig 2 represents these combinations using alphabetic characters from A to Z. A single alphabetic character denotes one unique combination of cardiac parameter values, and the same character across different folds (e.g., A) refers to the same combination of cardiac parameter values. A model trained on a limited range of EX parameter values (e.g., EX = 1, 2, 3) cannot generalize to completely unseen values (e.g., EX = 4, 5). To address this, we cyclically shuffled the data across the folds so that each fold contains multiple EX parameter values rather than just a single value. After shuffling, three of the five folds were used for training, one for validation, and one for testing.

The lengths of the input ECG signals were all *T* = 500. We optimized the proposed model by using the RAdam optimizer [35] with the parameters α=0.001 (learning rate), $\beta_{1}=0.9$, $\beta_{2}=0.999$, and $\varepsilon =10^{-8}$. The weight and bias parameters of *l*-th 1D-Conv layer (l∈{1,2,3,4,5}) in the encoder network were initialized to random values between $-\sqrt{1/k_{l}^{enc}}$ and $\sqrt{1/k_{l}^{enc}}$, where $k_{l}^{enc}$ is the product of the channel size and the kernel size of *l*-th 1D-Conv layer. The weight and bias parameters of *l*-th FC layer (l∈{1,2,3,4,5}) in the decoder networks were initialized to random values between $-\sqrt{1/k_{l}^{dec}}$ and $\sqrt{1/k_{l}^{dec}}$, where $k_{l}^{dec}$ is the input size of *l*-th FC layer. The batch size and the number of epochs in the training stage were 4096 and 20000, respectively. At the end of each epoch, we calculated the loss value for the validation data by using the weight and bias parameters obtained in that epoch, and the weight and bias parameters that achieved the lowest loss value were used to evaluate the model.

The estimation performance of the continuous and discrete cardiac parameters was evaluated by using the mean absolute error (MAE) and accuracy (ACC) defined as follows:


$$\begin{array}{cc} {MAE}_{i}=\frac{1}{N}\sum_{n=1}^{N}\left|\phi_{n,i}^{*}-\phi_{n,i}\right| & \;(i\in C),\\ {ACC}_{i}=\frac{1}{N}\sum_{n=1}^{N}1\left(\phi_{n,i}^{*},\phi_{n,i}\right) & \;(i\in D), \end{array}$$ (7)

where *N* is the number of data in the test dataset (i.e., *N* = 6144), $\Phi_{n}^{*}={\left(\phi_{n,i}^{*}\right)}_{i=1}^{15}$ is the *n*-th set of ground-truth cardiac parameters in the test dataset, $\Phi_{n}={\left(\phi_{n,i}\right)}_{i=1}^{15}$ is the set of cardiac parameters estimated by the proposed method from the *n*-th ECG in the test dataset, and 1(·,·) is an indicator function defined by


$$\begin{array}{c} 1\left(u,v\right)=\left\{ \begin{array}{cc} 1 & \left(u=v\right)\\ 0 & (u\neq v). \end{array}\right. \end{array}$$ (8)

In other words, the indicator function returns one if the estimated parameter value matches the ground-truth value, and zero otherwise. At the final step of each epoch, the validation loss was calculated using the latest trained model parameters. To evaluate the estimation performance, we used the trained model parameters that yielded the lowest validation loss.

## Results

**Fig 3:**
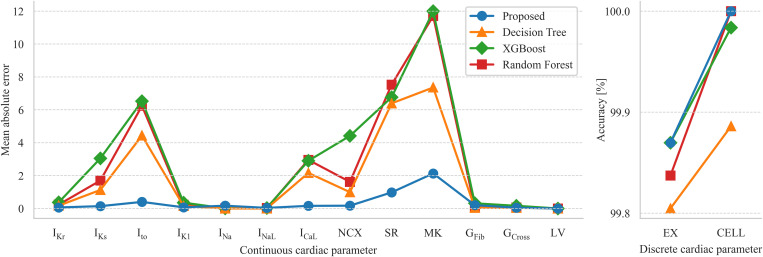
Evaluation results. The performance of the proposed method was measured by comparing the ground-truth cardiac parameters and the estimated cardiac parameters. The left line plot shows the mean absolute errors for the continuous cardiac parameters, and the right line plot shows the accuracies for the discrete cardiac parameters. The blue, orange, green, and red lines represent the performance of the proposed method, the decision tree, the XGBoost, and the random forest. The performance of the random forest is selected In this figure, we plot the results for the random forest with 100 trees and automatically determined depth. The horizontal axes in the plots indicate the kind of cardiac parameter.

The experimental results are shown in Table 3 and Fig 3. The left line plot represents the mean absolute errors for the continuous cardiac parameters, and the right line plot represents the accuracies for the discrete cardiac parameters. In addition, the performance of the proposed method was compared with that of tree-based models, including decision trees, XGBoost, and random forests. From scikit-learn [36] and XGBoost [37], we employed DecisionTreeRegressor, RandomForestRegressor, and XGBRegressor for estimating the continuous cardiac parameters, and DecisionTreeClassifier, RandomForestClassifier, and XGBClassifier for estimating the discrete cardiac parameters. As shown in Table 3 and Fig 3, the proposed method outperforms or is comparable to the tree-based models in estimating cardiac parameters. Although the mean absolute errors of the proposed method for the SERCA and MK parameters are slightly larger than those for the other continuous cardiac parameters, the errors are small compared with the range of values that the continuous cardiac parameters can take. In addition to accuracy, we report macro precision 𝒫, macro recall ℛ, and macro F1‑score ℛ for estimating the discrete cardiac parameters. As shown in Table 4, all models were able to estimate the discrete parameters almost correctly. One possible explanation of this results is that the synthetic ECGs are relatively simple, which may reduce the difficulty of the estimation of the discrete cardiac parameters.

**Table 3:** Evaluation results.

Model	n_estimators	max_depth	I_Kr_	I_Ks_	I_to_	I_K1_	I_Na_	I_NaL_	I_CaL_	NCX	SERCA	MK	G_Fib_	G_Cross_	LV	EX	CELL
Proposed	–	–	0.06	0.14	0.40	0.07	0.17	0.04	0.16	0.17	0.98	2.13	0.20	0.05	0.00	99.87	100.00
Decision Tree	–	None	0.17	1.13	4.43	0.15	0.00	0.00	2.16	0.99	6.39	7.36	0.04	0.04	0.00	99.80	99.89
XGBoost	100	None	0.37	3.05	6.53	0.35	0.01	0.03	2.90	4.42	6.76	11.99	0.31	0.16	0.00	99.87	99.98
Random Forest	10	10	0.31	5.80	10.56	0.31	0.01	0.02	5.79	4.55	8.85	19.79	0.10	0.16	0.00	99.84	100.00
Random Forest	100	10	0.31	5.93	10.54	0.31	0.01	0.03	5.91	4.19	8.85	19.79	0.17	0.18	0.00	99.85	100.00
Random Forest	10	None	0.23	1.64	6.17	0.21	0.00	0.02	3.04	1.69	7.51	11.82	0.05	0.05	0.00	99.89	100.00
Random Forest	100	None	0.24	1.70	6.24	0.22	0.01	0.03	2.95	1.62	7.53	11.70	0.05	0.06	0.00	99.84	100.00

**Table 4:** Precision, recall, and F1-score for the discrete cardiac parameters.

Model	n_estimators	max_depth	EX			CELL		
			𝒫	ℛ	ℱ	𝒫	ℛ	ℱ
Proposed	–	–	99.87	99.87	99.87	100.00	100.00	100.00
Decision Tree	–	None	99.80	99.80	99.80	99.89	99.89	99.89
XGBoost	100	None	99.87	99.87	99.87	99.98	99.98	99.98
Random Forest	10	10	99.84	99.84	99.84	100.00	100.00	100.00
Random Forest	100	10	99.85	99.85	99.85	100.00	100.00	100.00
Random Forest	10	None	99.89	99.89	99.89	100.00	100.00	100.00
Random Forest	100	None	99.84	99.84	99.84	100.00	100.00	100.00

We compared the performance of the proposed method and the tree‑based models for estimating the cardiac parameters. The n_estimator indicates the number of trees for Random Forest and the number of boosting rounds for XGBoost. The max_depth represents the maximum tree depth. When max_depth is set to None, no explicit constraint is imposed on it, and its value is automatically determined by the toolkits [36,37]. The evaluation metrics are the mean absolute error (Eq (7)) for the continuous cardiac parameters and the accuracy (Eq (8)) for the discrete cardiac parameters.

We evaluated the macro precision 𝒫, macro recall ℛ, and macro F1‑score ℱ of the proposed method and the tree‑based models for estimating the discrete cardiac parameters. The n_estimator indicates the number of trees for Random Forest and the number of boosting rounds for XGBoost. The max_depth represents the maximum tree depth. When max_depth is set to None, no explicit constraint is imposed on it, and its value is automatically determined by the toolkits [36,37].

**Fig 4:**
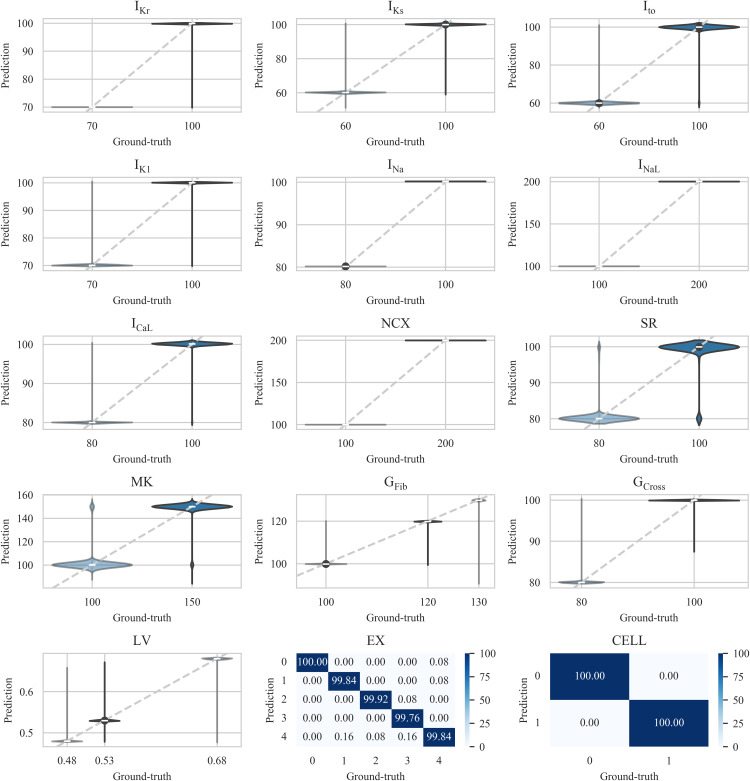
Distributions of the estimated cardiac parameters. The violin plots and heatmaps represent the distributions of the estimated continuous and discrete cardiac parameters around the ground-truth values.

To investigate the estimation errors in detail, Fig 4 shows the distributions of the estimated cardiac parameters around the ground-truth values. The violin plots are obtained from the estimated continuous cardiac parameters, and the heatmap plots represent the confusion matrices calculated from the ground-truth and estimated discrete cardiac parameters. Overall, these plots show that the proposed method successfully estimated both continuous and discrete cardiac parameters. The distribution of cardiac parameters with slightly large estimation errors (e.g., SR and MK) is bimodal with large and small peaks. The spread of the large peak indicates that the estimates of the proposed method deviate slightly from the correct values. The small peak indicates that the proposed model sometimes incorrectly estimates other values that the target cardiac parameter can take.

**Fig 5:**
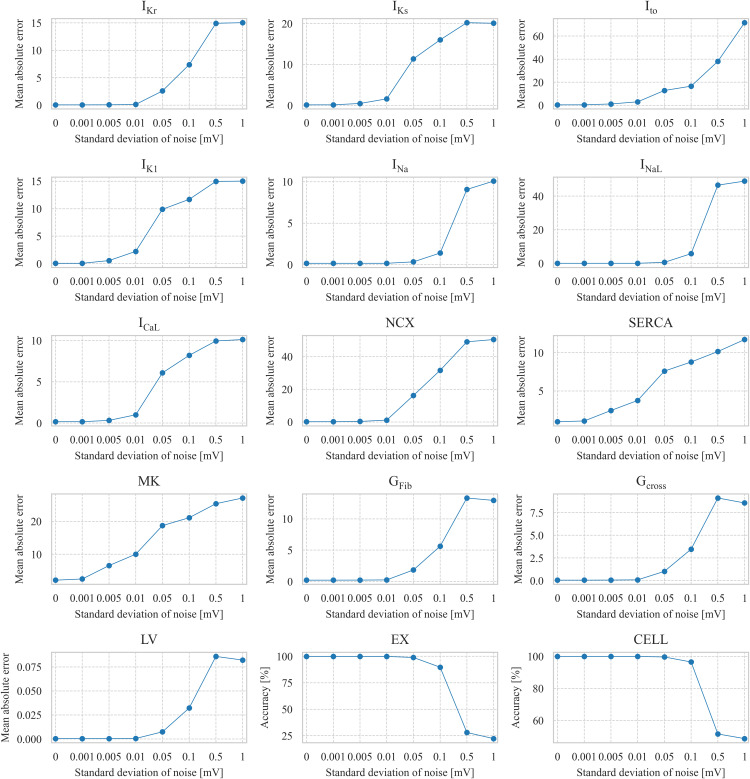
Evaluation results for noisy ECG signals. The performance of cardiac parameter estimation by the proposed method was measured on several ECG signals with different noise levels. In each graph, the horizontal axis represents the noise level, while the vertical axis represents the estimation performance.

**Fig 6:**
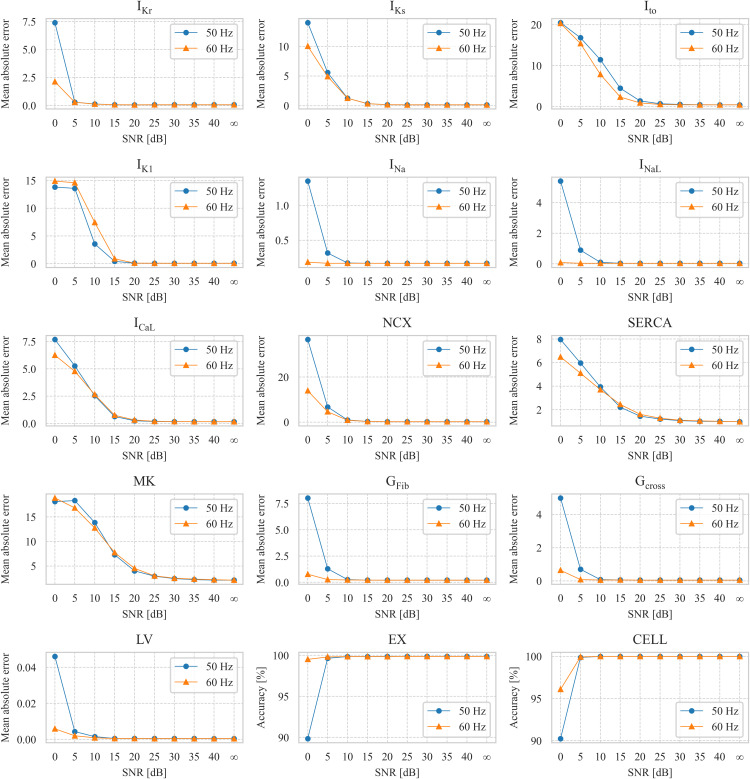
Evaluation results for ECG signals with power-line interference. We evaluate the proposed method on ECG signals with varying levels of power-line interference (PLI) noise. In each graph, the horizontal axis represents the signal-to-noise ratio (SNR) in dB, which indicates the noise level, and the vertical axis represents the estimation performance. SNR=∞ indicates that the ECG signal contains no PLI noise. The blue and orange lines represent the performance under 50 Hz and 60 Hz power-line interference, respectively.

We investigated the robustness of the proposed method against noise in ECG signals. Real ECG data often contains noise caused by various factors, such as electrical interference, body movement, and measurement errors. This noise can potentially reduce the accuracy of parameter estimation of the proposed method. Moreover, since the proposed method was trained only on synthetic data without any noise, it may be susceptible to noise, which could limit its applicability in practical settings. To examine this issue, we conducted experiments to evaluate the performance of the proposed method on noisy ECG signals. Since collecting real ECGs and corresponding cardiac parameters is challenging, we used synthetic ECGs with artificially generated noise. This study does not consider missing leads because we believe they should be prevented during recording. Noise $𝐖\in {\mathbb{R}}^{12\times T}$ was independently generated for each lead and each sample of the ECG signal $𝐗\in {\mathbb{R}}^{12\times T}$, following a standard normal distribution. The generated noise was then scaled according to the assumed noise level a∈ℝ in millivolts and added to the original ECG signal as 𝐗+a/(0.00488×1024)×𝐖. Here, dividing *a* by 0.00488 converts the noise level scale from millivolts (mV) to the amplitude scale of ECGs in the UT-Heart database. Furthermore, following the ECG amplitude transformation described in the dataset section, the noise level was divided by 1024. In this experiment, we examine cases where the noise level a∈{0.001,0.005,0.01,0.05,0.1,0.5,1}. The experimental results, shown in Fig 5, indicate that the performance gradually decreases as the noise level increases. According to [38], Sections 01.12.4.106.1 and 201.12.4.107.3 of IEC 60601-1-2-25 state that the noise level in ECG signals should not exceed 30 µV = 0.03 mV. When the noise level is relatively mild and does not exceed this threshold, the proposed method maintains a high level of performance. Additionally, we investigate the robustness of the proposed method against power-line interference (PLI) as a representative of ECG noises. In this experiment, the PLI noise synthesized as a sine wave is added to the ECG signal as $x_{t,c}+A\sin(2\pi ft/T)$ (c∈{1...,12}), where the noise frequency f∈{50,60} in Hz. We set the noise amplitude A∈ℝ such that signal-to-noise ratio in dB is one of {0, 5, 10, 15, 20, 25, 30, 35, 40}. As shown in Fig 6, the experimental results indicate that the proposed model is generally robust with respect to the PLI down to 15 dB SNR.

The experimental results and the scripts used to generate the result figures and tables are provided in S1 Materials.

## Discussion

The proposed method has the potential to help medical experts advance their fields of study. For example, by combining the parameters estimated by this method with the previous knowledge about these parameters, medical experts can learn more about the internal heart state, which is difficult to know from the morphological patterns of the ECG waveform alone. If the proposed method can identify abnormalities in key molecules associated with cardiac excitation and contraction from ECGs, these abnormalities can be treated with specific drugs or through genetic manipulation [39]. In addition, previously unknown but medically important ECG morphology patterns and relationships between ECGs and cardiac parameters can be discovered by comparing the estimated cardiac parameters with the ECG waveform. In the future, we must confirm whether the proposed method is actually helpful in these ways.

The experimental results show that the proposed model achieves estimation performance comparable to or better than that of other models for each cardiac parameter. In particular, it accurately estimates parameters such as I_Ks_, I_CaL_, SERCA, and MK, which are shown to be difficult for the tree‑based models to estimate. A similar trend is observed in our preliminary experiments comparing the proposed model with the baseline model using a single decoder. These results indicate that, unlike the tree‑based models that make threshold‑based decisions independently for each time sample in an ECG waveform, the proposed model can extract more informative representations by capturing relationships across samples within the waveform, thereby improving estimation performance. Additionally, the comparison in the preliminary experiments suggests that each decoder in the proposed model can extract parameter‑specific information, which further contributes to improving estimation performance.

This study has several limitations. First, it uses only synthetic ECGs. Since the proposed model was trained using only synthetic data, there is a potential risk of overfitting, and the dataset size may not be sufficient to prevent it. Although the ECGs used in this paper were generated from a wide range of cardiac parameter variations, the deterministically and synthetically generated ECGs might lack the noise and fluctuations observed in real ECGs. Investigating how well these synthetic ECGs cover the real-world variability of fluctuations and noise remains a subject for future research. Second, collecting the paired data of real ECGs and corresponding cardiac parameters is inherently and highly difficult because it requires direct measurement of physiological parameters from living subjects. Therefore, our study does not handle this problem.

Moreover, in the future, we will need to develop a practical method to verify whether the proposed method trained using only the data synthesized by UT-Heart works robustly with noisy and individually different ECGs obtained from real human bodies in a clinical environment. However, it is difficult to obtain real ECGs and the cardiac parameters required for such training and evaluation. To address this limitation, one possible approach is to use an ECG generator. If the ECG generator can perfectly reproduce ECG signals from cardiac parameters, then the performance of the proposed method can be indirectly measured by comparing the real ECG with the ECG reproduced by the ECG generator using the estimated cardiac parameters.

## Conclusion

We developed a method based on a DNN that takes ECGs as input and outputs the microscopic cardiac parameters that represent the internal heart state and are factors of ECG generation. We experimentally confirmed that our method can correctly estimate these cardiac parameters. To our best knowledge, this is a novel attempt to estimate multiple microscopic cardiac parameters simultaneously and comprehensively. As a future challenge, it remains to verify whether our method works correctly with real ECGs. Moreover, our method has the potential to be used for early diagnosis and in medical research. In particular, it may be useful in analyzing the relationship between the signals and parameters of various organs besides the heart.

## Supporting information

S1 Materials Experimental results and scripts used to generate the result figures and tables. (ZIP)
